# Screening for convergence insufficiency using the CISS is not indicated in young adults

**DOI:** 10.1136/bjophthalmol-2013-304533

**Published:** 2014-02-14

**Authors:** Anna M Horwood, Sonia Toor, Patricia M Riddell

**Affiliations:** 1 Infant Vision Laboratory, School of Psychology & Clinical Language Sciences, University of Reading, Reading, UK; 2 Orthoptic Department, Royal Berkshire Hospital, Reading, UK

**Keywords:** Diagnostic Tests/Investigation, Muscles

## Abstract

**Aim:**

This paper presents Convergence Insufficiency Symptom Survey (CISS) and orthoptic findings in a sample of typical young adults who considered themselves to have normal eyesight apart from weak spectacles.

**Methods:**

The CISS questionnaire was administered, followed by a full orthoptic evaluation, to 167 university undergraduate and postgraduate students during the recruitment phase of another study. The primary criterion for recruitment to this study was that participants ‘felt they had normal eyesight’. A CISS score of ≥21 was used to define ‘significant’ symptoms, and convergence insufficiency (CI) was defined as convergence ≥8 cm from the nose with a fusion range <15Δ base-out with small or no exophoria.

**Results:**

The group mean CISS score was 15.4. In all, 17 (10%) of the participants were diagnosed with CI, but 11 (65%) of these did not have significant symptoms. 41 (25%) participants returned a ‘high’ CISS score of ≥21 but only 6 (15%) of these had genuine CI. Sensitivity of the CISS to detect CI in this asymptomatic sample was 38%; specificity 77%; positive predictive value 15%; and negative predictive value 92%. The area under a receiver operating characteristic curve was 0.596 (95% CI 0.46 to 0.73).

**Conclusions:**

‘Visual symptoms’ are common in young adults, but often not related to any clinical defect, while true CI may be asymptomatic. This study suggests that screening for CI is not indicated.

## Introduction

Convergence insufficiency (CI) can result in asthenopia, headaches, blur, diplopia and other difficulties with close work. While it has been acknowledged as a common and treatable condition since the earliest days of orthoptic treatment, the scale of the problem is less clear. Reports of prevalence vary considerably between studies, with estimates varying between 0.1% and 8% appearing in the literature,^1^^–^6 depending on the population studied (children or adults), diagnostic criteria (near points anywhere between 6 and 12 cm) and whether cases are identified from community or screening studies or after seeking professional assessment for a troublesome symptom. Many of the most common symptoms (such as headache, loss of concentration, rereading or forgetting recently read text and feeling tired after close work) are not specific to CI.

Since the 1990s, the Convergence Insufficiency Treatment Trial Group (CITTG) has been studying different methods of treating CI in the course of which they developed the Convergence Insufficiency Symptom Survey (CISS) questionnaire as a validated method of quantifying and monitoring symptoms in CI.^2^ In a masked study of the test's validity, the group found that a score of ≥16 could reliably distinguish children with symptomatic CI from those with normal binocular vision,^7^ though more recent studies have questioned this value^8^ ^9^ and the adult cut-off is now recommended as ≥21.^10^

We have been studying a group of students for a study on the effects of different orthoptic exercises and needed to exclude those with any significant visual problems. As the CISS appeared to have been well validated as a method to quantify CI, we hoped that it would also help us identify and exclude individuals with the condition, although we accept it was not designed as a screening tool. We expected at least to find a fairly close correlation between symptoms and clinical signs.

Others have expressed concerns about some of the methods used by the CITTG.^11–14^ We also felt that some of the questionnaire items were very general and arguably typical of a young adult or student lifestyle, so in this study we also took the opportunity to explore these issues.

## Methods

### Recruitment

The University of Reading School of Psychology Participant Database and University email lists were used to recruit undergraduate and postgraduate students as part of a study investigating the effect of orthoptic exercises on convergence and accommodation^15^ ^16^ (in press). They were to be offered up to £20 if they were accepted onto, and completed, the study. The majority of participants were first and second year psychology students, with a smaller proportion being undergraduates and postgraduates from other science disciplines. Overall, 95% spoke and read English as their first language. All participants were undertaking a significant academic workload.

As a primary screening criterion prior to being able to volunteer, the participants were asked to confirm that they considered themselves to ‘have normal eyes apart from weak glasses (±4.0D)’ since we wanted to research typical responses in asymptomatic participants. Current or previous strabismus, orthoptic or vision therapy, eye examinations beyond regular refraction checks, seeking treatment for visual symptoms in the past, or taking part in previous experiments in our laboratory were used as other exclusion criteria. Only if they passed this primary screening were they offered an appointment for initial assessment.

### CISS questionnaire

The CISS questionnaire was emailed to the participants prior to their first visit to the lab and they were asked to fill it in and bring it with them when they came, before they had any experience of our testing. They were told it was just to help us confirm the absence of significant visual symptoms in a quantifiable fashion.

**Figure 1:**
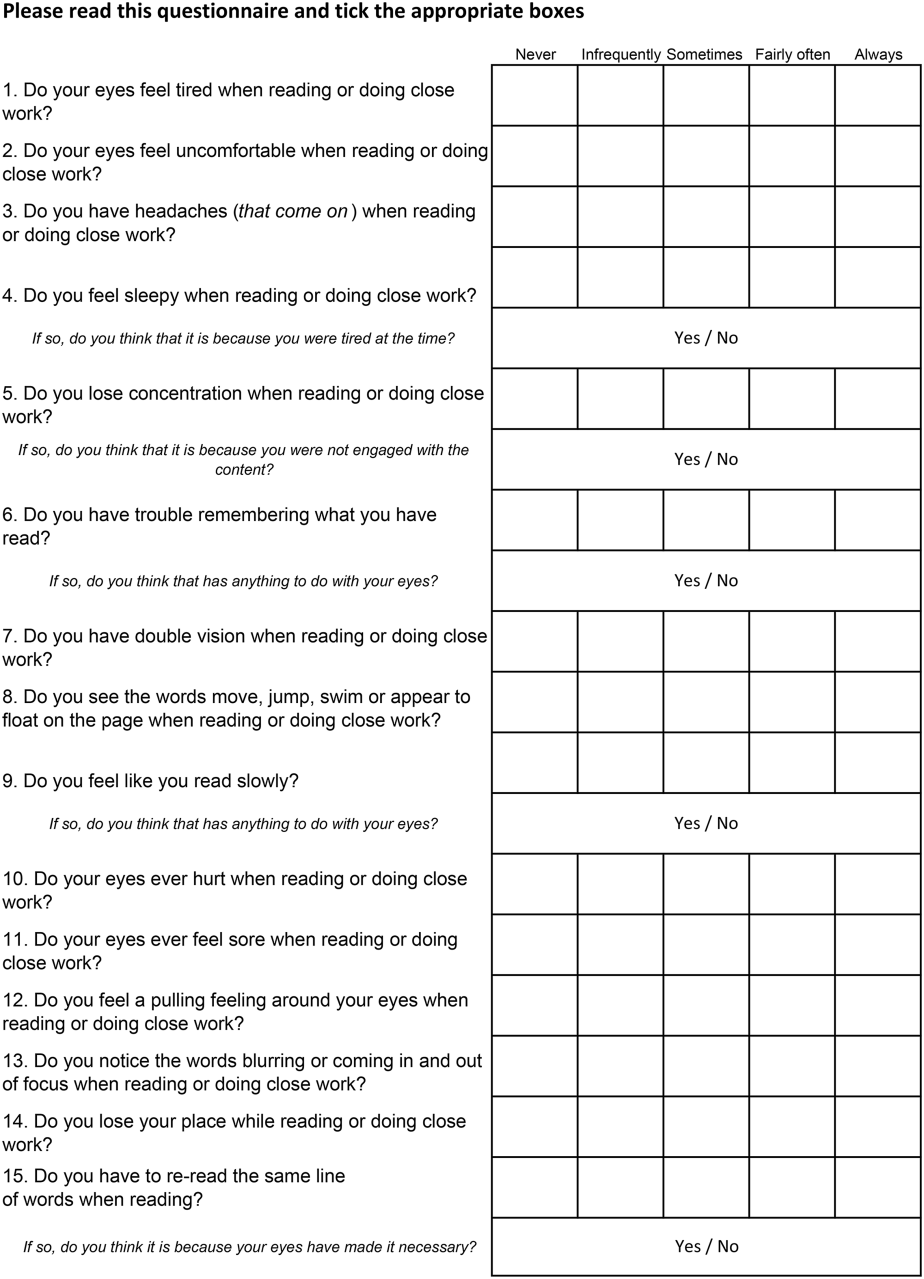
The convergence insufficiency symptom survey.

Participants were following a typical student lifestyle, sometimes involving doing difficult, very technical or tedious assignments when tired, involving extended close work, and working to deadlines. We expected that some of the subjects may have attention deficit hyperactivity disorder, dyslexia or other literacy difficulties that might slow their reading. These factors could be expected to lead to reports of issues with fatigue, concentration, comprehension, memory or reading speed that would be unrelated to visual problems. Because we considered that five of the 15 CISS items could relate to non-ocular difficulties, supplemental questions were asked after those five questions to ascertain whether the symptoms were considered to be ocular by the subject (see figure 1).

The CISS questionnaire was scored as usual on a 5-point scale with 0=‘never’ and 4=‘always’. If the answer to the supplemental questions suggested a non-ocular cause, the question was rescored as 0 to give an additional adjusted score.

### Clinical testing

The participants were tested while wearing their current refractive correction. The battery of orthoptic tests included: corrected visual acuity, cover testing, ocular motility assessment, convergence (NPC) and accommodation near points (NPA), prism fusional ranges to blur, diplopia and recovery, vergence and accommodative facility, TNO stereotest and prism cover test for measurement of heterophoria. Participants with a manifest or intermittent strabismus, corrected visual acuity worse than 0.1 logMAR, anisometropia >0.75D in any meridian, a heterophoria >8 prism dioptres exophoria or 1 prism dioptres esophoria at any distance, or stereopsis worse than 120 s of arc TNO were excluded from this study. CI was diagnosed if NPC was ≥8 cm from the bridge of the nose and the prism fusion range failed Sheard's criterion (fusional vergences being more than double the heterophoria^17^) or was less than 12Δ base-out (BO) blur/15 Δ BO break. We defined poor convergence as ≥8 cm from the nose rather than the very strict ≥6 cm criteria used by the CITTG because most criteria use 8–10 cm.^18–21^ We chose 8 cm as being in the middle of this range. We used the UK definition of CI, which includes poor convergence without concurrent near exophoria,^22^ and we excluded large near exophorias so as to consider pure convergence problems.

### Analysis

Sensitivity and specificity, positive and negative predictive values for the CISS to detect CI were calculated. Receiver operating characteristic (ROC) curves were plotted. The closer a ROC curve comes to the 45° diagonal of the ROC space and the smaller the area under the curve, the lower the ability of the test to accurately diagnose a condition.

## Results

In all, 171 students completed the questionnaire and attended the lab for testing. All were between 18 and 26 years of age and 144 were women. Four volunteers were excluded after orthoptic assessment due to previously undiagnosed significant heterophoria or microstrabismus, leaving 167 participants. Two participants had a prior diagnosis of dyslexia.

Overall, 17 subjects (10.2%) showed clinical signs of CI according to the definition above and 41 subjects (24.5%) had a high CISS score. Thus, 52 participants (31.1%) had either a high CISS score or showed clinical signs of CI (see table 1).

**Table 1:** Numbers (and percentage of the whole group)

	Clinical CI		No CI	
High CISS score (≥21)	6 (3.6%)	True +ve	35 (20.9%)	False +ve
Low CISS score (<21)	11 (6.5%)	False −ve	116 (69.5%)	True −ve
High CISS score (≥21) adjusted	2 (1.1%)	True +ve	13 (7.7%)	False +ve
Low CISS score (<21) adjusted	15 (8.9%)	False −ve	137 (82%)	True −ve

CI, convergence insufficiency; CISS, Convergence Insufficiency Symptom Survey.

### Unadjusted scores

Median CISS score for the whole group was 15.4 (range 0–40, IQR 9–20). A total of 41 participants (24.5%) scored ≥21, a ‘high’ score according to the CITTG criterion. At least 80% (80%–91%) of participants felt that symptoms reported on items 4 (feeling sleepy), 5 (losing concentration), 6 (problems remembering), 9 (slow reading) and 15 (rereading lines) had nothing to do with their eyes.

Of the 17 clinically diagnosed CIs, only six participants (35.9%) returned a high score (median 26) while 11 returned a low score (median 11). Mean NPC was 9.7 cm for the high-symptom scoring CI group and 9.3 cm for the low-scoring CI group. In all, 35 of the 150 (23.3%) without any clinical signs of CI returned a high CISS score ≥21 (median 27, range 21–40).

**Figure 2:**
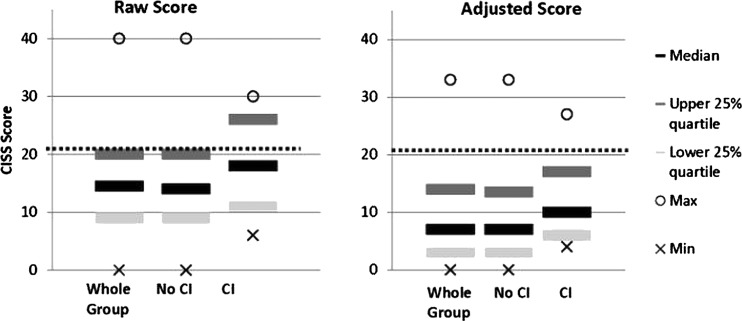
Distribution of Convergence Insufficiency Symptom Survey (CISS) scores in the whole group, no convergence insufficiency (CI) and CI groups. Dotted line indicates CISS diagnostic cut-off of 21.

Figure 2 illustrates the distribution of scores in the whole group, and then divided into non-CI and CI groups.Sensitivity of the CISS for CI in this sample was 37.5%. Specificity was 76.8%. Positive predictive value was 14.6% and negative predictive value was 92.1%.

**Figure 3:**
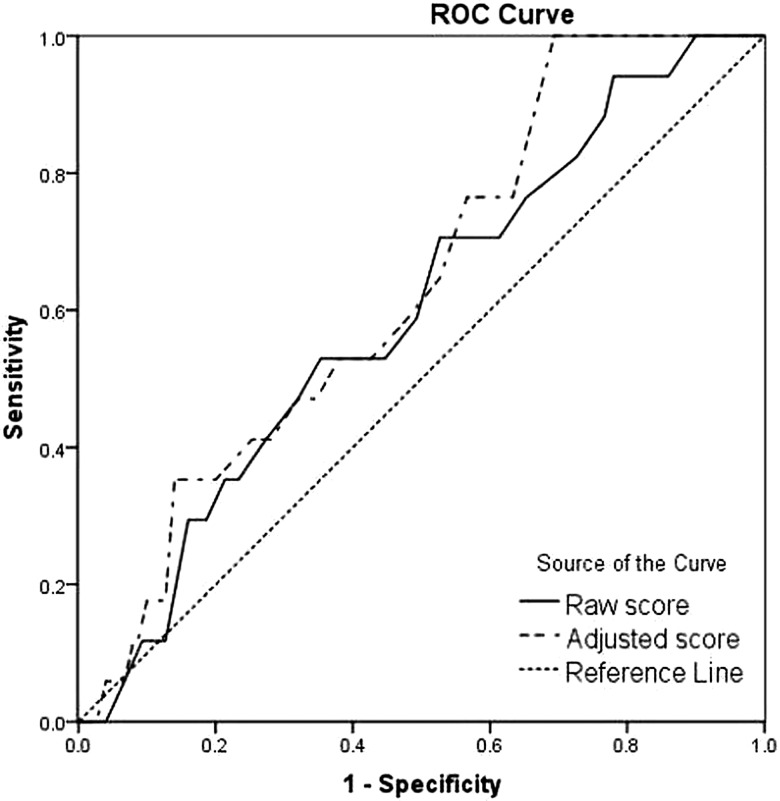
Receiver operating characteristic (ROC) plot of Convergence Insufficiency Symptom Survey (CISS) score in the detection of clinical convergence insufficiency using the raw CISS score and the adjusted score.

The ROC curve is illustrated in figure 3. A test which is a poor predictor of disease shows a curve close to the diagonal (area of 0.596). The area under the curve was 0.596 (95% CI 0.46 to 0.73).

When the analysis was repeated on scores after adjustment for non-ocular causes, results were little better. Median CISS score reduced as expected to 7 (range 0–33, IQR 3–14), but 15 (8.9%) participants still had a CISS score ≥21 (now from 10 instead of 15 items), of which only 2 (13.3% of these) had genuine CI; so, 13 (7.7% of all participants) without any other clinical signs of CI still returned a high CISS score. Mean NPC was 9.0 cm for the two high-symptom scoring CI participants, and was slightly worse for the remaining 15 with CI but ‘low’ symptom scores (9.5 cm).

Sensitivity of the adjusted CISS scores for CI was 11.7%, specificity was 91.3%, positive predictive value was 13.3% and negative predictive value was 90.1% The area under the ROC curve improved slightly to 0.636 (95% CI 0.51 to 0.76).

All volunteers with clinically diagnosed CI were asked whether they were sure they considered themselves to be asymptomatic and had no difficulties with their workload. Eight cases showed more severe CI with convergence beyond 10 cm and a reduced near BO fusion range of <15Δ (so within most definitions of CI, and which most orthoptists or optometrists would typically treat). All 17 were offered a course of treatment to either treat or prevent symptoms, but all declined.

## Discussion

This study demonstrates that symptoms often associated with CI are also common in young adults without clinical signs of poor convergence, and also that the majority of subjects with the clinical signs of CI (reduced convergence and fusion range) have no symptoms. Somewhat similar findings in children were described by Ip *et al.*^23^

The CISS was designed to be used as a tool to serve as a measure of treatment effect in clinical trials, with different scores used to distinguish symptomatic CI from those with normal binocular vision in children (≥16) and adults (≥21).^8^ ^10^ This study clearly demonstrates that the CISS questionnaire cannot be used as a screening tool in ‘asymptomatic’ non-clinical populations because of poor sensitivity and a high false positive rate. Use of the CISS has been validated in studies on treatment trials of children^7^ and adults^10^ but some researchers do not agree that the CISS is a good tool even for that purpose.^11–13^ While the CISS was never designed as a screening tool, it is readily available online, possesses considerable face validity and appears to be being used as such. The authors have personally been asked to review papers where it has been used by non-eye care researchers in areas such as education and epidemiology, but such use risks significant over-referral for suspected vision problems. There appears no additional value in attempting to adjust scores for possible ‘non-ocular’ reasons for symptoms.

It may be a useful method of monitoring symptoms in established, symptomatic CI, or for the research contexts for which it was designed, but if it had been used to screen our student population with no previous history of problems, 25% of students would have been referred for further investigation, but even so, 65% of the true CIs would have been missed. Even after adjusting for non-ocular causes of symptoms 9% of students would have been referred, but 88% would have been missed.

We found that 10% of our asymptomatic sample had what some ocular professionals would define as ‘clinical convergence problems’, but because we excluded significant heterophorias, and because students who already suspected they might have a problem may have excluded themselves from volunteering, this figure cannot be used to give any generalisable prevalence data. We accept that we only saw cases of mild CI because individuals with more severe problems are likely to seek a professional opinion and in this case would have been excluded during the selection process; there may be clearer links among symptom score, perception of a problem and clinical findings in more severe cases.

We considered whether offering payment for participation might have skewed our results. We specifically asked for people ‘who considered their eyes normal’ and listed conditions that would preclude participation. If money had been an incentive to play down any symptoms in order to be able to participate, we would have expected to find low symptom levels reported, despite clinical signs of CI, but the opposite was the case; symptoms were common, but did not appear to be considered abnormal or troublesome; neither did they relate strongly to clinical signs.

This study raised the issue of what constitutes a ‘problem’. Porcar and Martinez-Palomera^24^ found a high prevalence of CI and other binocular problems in university students, with an overall prevalence of 32.3%. Although 31% of our sample had either a high CISS score or clinical CI, none of them thought they had a problem with their eyes, and all those offered treatment declined it. So who is right, the professional or the patient?

Some professionals might argue that early detection and treatment of a mild asymptomatic problem might prevent more severe difficulties later, or that people might not realise how much better things could be if they had better convergence or fusion; but it could equally be true that being a student can be challenging and a certain amount of ‘symptoms’ are a normal part of a typical lifestyle, often unrelated to eye signs. Mild CI also does not seem to be a problem for many people. Concentration may wane; content may be complex and often boring; print may be small, indistinct and dense, and many people have diagnosed or undiagnosed literacy issues; and studying often has to be carried out under suboptimal circumstances such as when tired, in poor lighting or when stressed. Eye exercises will not change these factors, may add another level of stress, and might incur significant financial cost either to the ‘patient’ or to publicly funded healthcare services. The current protocol recommends that CI should be treated if visual symptoms associated with close work cause an individual to seek professional advice. These data suggest screening for CI using the CISS is not indicated in young adults.
